# The Thai version of the PSS-10: An Investigation of its psychometric properties

**DOI:** 10.1186/1751-0759-4-6

**Published:** 2010-06-12

**Authors:** Nahathai Wongpakaran, Tinakon Wongpakaran

**Affiliations:** 1 Department of Psychiatry, Faculty of Medicine, Chiang Mai University, Chiang Mai, 50200, Thailand

## Abstract

**Background:**

Among the stress instruments that measure the degree to which life events are perceived as stressful, the Perceived Stress Scale (PSS) is widely used. The goal of this study was to examine the psychometric properties of a Thai version of the PSS-10 (T-PSS-10) with a clinical and non-clinical sample. Internal consistency, test-retest reliability, concurrent validity, and the factorial structure of the scale were tested.

**Methods:**

A total sample of 479 adult participants was recruited for the study: 368 medical students and 111 patients from two hospitals in Northern Thailand. The T-PSS-10 was used along with the Thai version of State Trait Anxiety Inventory (STAI), the Thai Version of the Rosenberg Self-Esteem Scale (RSES), and the Thai Depression Inventory (TDI).

**Results:**

Exploratory Factor Analysis (EFA) yielded 2 factors with eigenvalues of 5.05 and 1.60, accounting for 66 percent of variance. Factor 1 consisted of 6 items representing "stress"; whereas Factor 2 consisted of 4 items representing "control". The item loadings ranged from 0.547 to 0.881. Investigation of the fit indices associated with Maximum Likelihood (ML) estimation revealed that the two-factor solution was adequate [*χ*^2 ^= 35.035 (*df *= 26, N = 368, *p *< 0.111)]; Goodness-of-Fit Index (GFI) = 0.981; Root Mean Square Residual (RMR) = 0.022; Standardized Root Mean square Residual (SRMR) = 0.037, Comparative Fit Index (CFI) = 0.989; Normed Fit Index (NFI) = 0.96, Non-Normed Fit Index (NNFI) = 0.981, Root Mean Square Error of Approximation (RMSEA) = 0.031. It was found that the T-PSS-10 had a significant positive correlation with the STAI (*r *= 0.60, *p *< 0.0001), and the TDI (*r *= 0.55, *p *< 0.0001); and was significantly negatively correlated with the RSES (*r *= -0.46, *p *< 0.0001, N = 368). The overall Cronbach's alpha was 0.85. The ICC was 0.82 (95% CI, 0.72 and 0.88) at 4 week-retest reliability.

**Conclusions:**

The Thai version of the PSS-10 demonstrated excellent goodness-of-fit for the two factor solution model, as well as good reliability and validity for estimating the level of stress perception with a Thai population. Limitations of the study are discussed.

## Background

Lazarus proposed that for an event or situation to be considered stressful, it must be perceived as stressful via perceptual processes [1,2]. The impact of stress depends upon the individual's perception of it and the resources available to control it [1]. Among the stress instruments that measure the degree to which life events are perceived as stressful, the Perceived Stress Scale theoretically corresponds to the model of stress proposed by Lazarus.

The Perceived Stress Scale (PSS), developed by Cohen, Kamarak, and Mermelstein[3], is a self-assessment tool created to measure the degree to which life events are appraised as stressful[3]. It addresses an individuals' perception of their lives and asks them to assess aspects of their life as unpredictable, uncontrollable, and overloading. Even though the PSS is focused more towards global, as opposed to life-event stressors, it is sensitive to chronic stress stemming from life circumstances[3].

The PSS has been used in various clinical circumstances, settings and cultures, and has been translated into 17 languages including Thai[4]. It has been used to predict outcome of response to antidepressant treatment in depressed patients with co-morbid personality disorders [5], as an interactional variable with dysfunctional attitudes in predicting depressive symptom severity following antidepressant treatment in patients with chronic depression[6], and as the link factor between food consumption frequency, perceived stress and depression[7].

Originally, the PSS had 14-items, and then it was revised to 10-items and 4-items respectively. Researchers have found good psychometric properties with regard to validity and reliability for all versions of the PSS scales, except for the latest version, or the PSS-4[8].

Despite the existence of a Thai version of the scale, its psychometric properties have not been examined. Therefore, the goal of this study was to investigate the psychometric properties of the Thai version of the PSS. We chose the PSS-10, which is a brief, easy-to-use version with equivalent psychometric properties to the PSS-14, as advised by Cohen and William[3]. We aimed to examine the validity and reliability of the Thai version of the PSS in two different samples: one sample consisting of students, and another of patients. Internal consistency, test-retest reliability, the concurrent validity, and the factorial structure of the scale were all tested.

## Methods

### Subjects

This study was approved by two independent ethics committees. A total of 479 adult participants were recruited for the study. Three hundred and sixty-eight medical students in years 1 to 5 of a medical school in Chiang Mai, Thailand, voluntarily participated in the study. In pre-clinical years, psychological self-assessment is a part of a learning activity in the subject "General Medical Professional Development (MPD)". In clinical years, this same process is used in order to monitor and provide mental health services for students who may need them. The student participants ranged in age from 19 to 25 (M = 20.84 years, SD = 0.95 years), and 58 percent were female.

In the clinical sample, 111 patients being treated for depression were recruited from the psychiatric outpatient clinics at a university hospital in Chiang Mai, and a general hospital in Lampang, Thailand. The clinical participants ranged in age from 18 to 74 (M = 34.20 years, SD = 12.41 years), and 63 percent were female.

### Procedures

The students were informed about the study after a class by a research assistant, who was not associated with the class. Interested students were provided with a take-home pack containing an information sheet, questionnaires, and an informed consent form. Students were also asked to choose/remember a code name (e.g. a number combination), to maintain students' anonymity. Informed consent was obtained by the initial testing and 4-week retesting. Individually, students later returned the completed questionnaires and the completed informed consent form to the research assistant, who promptly separated the informed consent form from the anonymous data.

Approximately four weeks after the initial testing, the same research assistant notified students via student announcement that retesting was due. Retest questionnaire distribution mirrored that of the initial testing. Student questionnaires without an accurate code name, or without an initial testing mate, were discarded. No compensation was given for their participation.

In the clinical sample, intake nurses screened the patients, and those assessed as potentially depressed were offered patient information sheets and informed consent forms by a research assistant. Two psychiatrists assisted with the study (one at each site) and used the Thai version of the Mini International Neuropsychiatric Interview (M.I.N.I.) as a criterion standard for identifying the presence or absence of Major Depressive Disorder (MDD)[9]. M.I.N.I. was subsequently administered on patients the day of data collection.

Interested patients were provided with a pack containing a patient information sheet (PIS), questionnaires, and an informed consent form. They were also asked to choose/remember a code name (e.g. a number combination) to maintain their anonymity. Informed consent included initial testing and 4-week retesting. After returning the completed informed consent forms individually to the research assistant, patients were asked to complete the questionnaires in an allocated room and then return the completed questionnaires to the research assistant. In the later appointment (approximately four weeks after the initial testing), the same research assistant notified patients about retesting. Retest questionnaire distribution mirrored that of the initial testing. Patient questionnaires without accurate code names, or without an initial testing mate, were discarded. Test-retest analysis was performed only on the student sample.

### Instruments

#### Development of Thai version of Perceived Stress Scale-10

The PSS-10[8] measures the degree to which one perceives aspects of one's life as uncontrollable, unpredictable, and overloading. Participants are asked to respond to each question on a 5-point Likert scale ranging from 0 (never) to 4 (very often), indicating how often they have felt or thought a certain way within the past month. Scores range from 0 to 40, with higher composite scores indicative of greater perceived stress. The PSS-10 has demonstrated good internal consistency[8].

In this Thai version, the authors translated the original version into Thai language with cultural adaptations, and then this was back-translated by an English-Thai bilingual school teacher, who had no knowledge of the wording of the original English version of the PSS-10. The two versions were then compared item-by-item and minor discrepancies were addressed and corrected by a consensus of the authors and the school teacher. Thirty individuals including relatives of patients, psychiatric patients and students (in different courses and years), who were not participating in the study, were asked to complete the Thai version PSS for a pilot study. Additional grammatical errors and misspellings were subsequently corrected. The revising procedure was performed once with acceptable results before field-testing began.

#### Thai version of State Trait Anxiety Inventory (STAI)

STAI is a common trait and state anxiety scale developed by Spielberger et al [10]. It is a 20-item instrument that measures trait and state anxiety. Respondents rate their anxiety on a 4-point scale ranging from 1 (not at all) to 4 (mostly). Higher scores are associated with greater feelings of anxiety. An example of a response is "I feel secure". STAI was translated into the Thai language and tested for its reliability by T. Nonthasak (In Techakomol W. [11]). In a previous study, Roberti et al found a high correlation between STAI and the PSS-10, indicating concurrent validity of the scales (r = 0.83, *p *< 0.0001)[12]. In the current study, the Thai STAI had a satisfactory internal consistency (Cronbach's alpha = 0.89).

#### Thai Version of the Rosenberg Self-Esteem Scale (RSES)

The RSES [10] was also utilized to examine the concurrent validity with the T-PSS-10. The RSES is a 10-item questionnaire with a 4-point Likert scale, ranging from "strongly agree" to "strongly disagree." Higher scores are associated with higher levels of self-esteem. An example question is "On the whole, I am satisfied with myself." Researchers have shown that unstable levels of self-esteem along with high levels of perceived stress can result in the development of depression [13]. The Thai-RSES had acceptable internal consistency in the current study sample (Cronbach's alpha = 0.87).

#### Thai Depression Inventory (TDI)

The TDI was developed by Lotrakul and Sukanich[14], and is a 20-item Thai instrument that measures the severity of depression. Respondents used a 4-point Likert scale ranging from 1 (no symptom) to 4 (most severe), such that higher scores are associated with greater feelings of depression. This scale showed a high correlation with the Hamilton Depression Rating Scale (HDRS) (*r *= 0.72, *p *< 0.0001). The severity of depression is scored as follows: ≤ 20 no depression, > 21 mild depression, > 35 major depression, > 40 severe major depression. A previous study showed that the PSS-10 had a moderate correlation with the Beck Depression Inventory (BDI) (*r *= 0.76, *p *< 0.001)[15]. The TDI had acceptable internal consistency in the current study sample (Cronbach's alpha = 0.89).

## Results

### Descriptive statistics

The mean score for the T-PSS-10 was 13.53, SD 4.56 for the whole student sample, and 13.99 (SD 4.27) for the whole clinical sample.

Out of the total 479 participants, 64 were categorized as having major depression. In particular, 19 out of the student sample (n = 368) met the clinical cut-off for major depression on the TDI, whilst 45 out of clinical sample (n = 111) were assessed as having major depression by psychiatrists using the M.I.N.I.

There was a significant difference in the T-PSS scores between the groups "with" (N = 64) and "without" (N = 415) major depression (mean ± SD = 17.33 ± 3.8 vs. 13.14 ± 4.3, *t *= 7.11, *df *= 477, *p *< 0.0001, Mean difference = 4.19, 95% Confidence Interval of the Difference 3.03-5.35). No significant differences in the T-PSS-10 scores were found in relation to age across both groups. Regarding age, there was a significant positive relationship between age and the T-PSS-10 scores in both student and clinical samples (*r *= 0.13, *p *= 0.013 and *r *= 0.28, *p *= 0.003 respectively). Notably, an opposite pattern was reported in the original version of the scale[8].

### Reliability

The internal consistency of the scale was good with a Cronbach's Alpha of 0.84 in the student group and 0.80 in the clinical group. At 4 week-retest reliability, the intra-class correlation coefficient (ICC) was calculated and found to be good for the student sample (N = 242, ICC = 0.83, 95% CI = 0.722-0.881).

### Factor analysis

#### Factor structure

To add more valued information, the two samples were independently analysed. We chose to use the larger sample size of the student group (N = 368) for exploring a confirmatory analysis of the model, whereas the clinical sample (N = 111) was analyzed by EFA. Prior to conducting the EFA, the sample data were screened to make sure that no assumptions were violated. Sampling adequacy was good with a Kaiser-Meyer-Olkin (KMO) value of 0.88 and Bartlett's test of sphericity was significant (*p *< 0.0001)[16]. To identify the factor structure, an EFA using a maximum likelihood (ML) method for extraction with eigenvalue greater than 1 was employed. We used an oblique rotation to account for correlations between the two factors (*r *= 0.51). EFA yielded eigenvalues of 5.050, 1.597, and 0.712 for the first three components. The first two factors with eigenvalues of 5.050, and 1.597 accounted for 50.50 percent and 15.97 percent of the variance respectively. Factor 1 consisted of 6 items representing "stress" (Items 1, 2, 3, 6, 9, and 10) and yielded Cronbach's alpha 0.90, M = 5.25, SD = 4.9; whereas Factor 2 consisted of 4 items representing "control" (Items 4, 5, 7, and 8) and yielded Cronbach's alpha 0.83, M = 5.10, SD = 3.77. The item loadings ranged from 0.547 to 0.881.

#### Confirmation of the Exploratory Model

We used CFA to determine the fit and number of factors to retain in the previously identified two-factor model. Lisrel 8[17] was used to compare the observed structure with the structure proposed in the theoretical model. The ML estimation method was used to test the covariance matrix to determine how well the model fit the sample data. In investigating the fit indices associated with ML estimation, the two-factor solution was shown to be adequate, [*χ*^2 ^= 35.035 (*df *= 26, N = 368, *p *< 0.111)]; Goodness-of-Fit Index (GFI) = 0.981; Non-Normed Fit Index ( NNFI) = 0.981; Normed Fit Index (NFI) = 0.960; Comparative Fit Index (CFI) = 0.989. Root Mean Square Residual (RMR) = 0.022; Standardized Root Mean square Residual (SRMR) = 0.037; Root Mean Square Error of Approximation (RMSEA) = 0.031. Results from the Confirmatory Factor Analysis (CFA) and ML fit indices suggest the presence and retention of a PSS-10 two-factor model, representing a reasonable approximation to the population.

#### Concurrent Validity

As expected, the T-PSS-10 scores were correlated with other measures including the State Trait Anxiety Inventory (STAI) (*r *= 0.60, *p *< 0.0001), the Thai Depression Inventory (TDI) (*r *= 0.55, *p *< 0.0001), but significantly negatively correlated with the Rosenberg Self-Esteem Scale (RSES) (*r *= -0.46, *p *< 0.0001, N = 368).

## Discussion

Overall, the psychometric data presented support the validity and reliability of the Thai PSS-10. The internal consistency of the scale was high and the reported correlations with measures of anxiety, depression, and self-esteem supported the concurrent validity of the scale. The mean scores were highly comparable to the original scale by Cohen[8]. The mean score in the group with MDD was higher than that of the non-major depression group ("no depression" to "less than MDD"). This may be explained by the association between the level of depression and level of perceived stress [18,19].

With regard to the PSS-10 structure, researchers have found that it has 2 related latent factors[8,12]. Compared to Cohen's original analysis, two factors yielded the eigenvalues of 3.4 and 1.4, which accounted for 48.9% and 14.5% of the variance respectively[8]. Roberti et al[12] yielded the eigenvalues of 5.07 and 1.12, accounting for 50.66% and 11.23% of the variance respectively. For a comparison between Roberti et al's results and the present study see Table 1. According to Roberti et al[12], factor analysis extracted two factors consisting of six items in Factor 1 and four items in Factor 2 that explained 61.9% of the variance. Factor 1 represents "Stress" or "Perceived Helplessness" as described by Roberti et al[12], whereas Factor 2 represents "Control" or "Perceived Self-Efficacy." Short-term stability of the PSS had been demonstrated. The results of the factor loadings on each item between both studies were comparable.

**Table 1:** Comparing the descriptive Statistics and Factor Analytic Findings of the PSS-10 between Roberti et al's study and the present study

Item	Factor I		Factor II		Communality	
	
	**Roberti et al's**[12]	The present study	**Roberti et al's**[12]	The present study	**Roberti et al's**[12]	The present study
1	0.81	0.881	-0.17	-0.02	0.46	0.76
2	0.64	0.867	0.17	0.045	0.56	0.79
3	0.71	0.663	-0.01	-0.107	0.44	0.38
6	0.63	0.547	0.02	0.162	0.4	0.42
9	0.53	0.759	0.15	0.035	0.43	0.61
10	0.67	0.801	0.15	0.054	0.58	0.69
4	-0.05	-0.054	0.79	0.638	0.49	0.37
5	-0.04	-0.006	0.83	0.761	0.54	0.57
7	0.00	0.381	0.70	0.798	0.46	0.49
8	0.21	0.516	0.57	0.868	0.52	0.78
Eigenvalue	5.07	5.05	1.12	1.60	Total	Total
%variance	50.66	50.53	11.23	15.97	61.9	66.47
M	12.09	5.25	6.06	5.10		
SD	4.72	4.9	2.20	3.77		
Cronbach's alpha	0.85	0.90	0.82	0.83		

Roberti et al's study[12] N = 285; The present study N = 111

Factor analysis yielded mostly similar results to those found by Roberti et al[12], except for the mean score and standard deviation of each factor. This is because the average scores in this student sample (mean = 13.53) were lower than those in Roberti et al's sample (mean = 18.15)[12]. Concerning item loadings, items 7 and 8 had high loadings (> 0.3) on the other factors in the present study, especially Item 8. These same results were found in both studies. In terms of fit statistics, Roberti et al's[12] found that the PSS-10 revealed an adequate two-factor solution: *χ*^2 ^= 121.78 (*df *= 34, N = 281, p < 0.001); goodness of fit index = 0.926, Root Mean Square Residual = 0.039, Comparative Fit Index = 0.931. The T-PSS-10 demonstrated a slightly better goodness-of-fit for the two-factor solution model compared to that of Roberti et al's[12]. This may be due to the larger sample size in the present study.

A previous study has shown that the PSS-10 has concurrent validity with a number of other measures including the WHO Wellbeing Index (WB), the State Trait Anxiety Inventory (STAI), the Beck Depression Inventory (BDI), the Patient Health Questionnaire (PHQ), the Shortened Cook-Medley, and the Hostility Scale (HS) (*r *= -0.77, 0.81, 0.76, 0.64, and 0.52 respectively)[15]. This was confirmed by a moderate correlation with other measures in the current study (*r *= -0.46, 0.55, and 0.60 for RSES, TDI and STAI respectively; *p *< 0.0001).

The overall Cronbach's alpha of the T-PSS-10 was 0.85. Compared to other studies by Cohen[8] Roberti[12] and Stauder[15] where reliability coefficients ranged from 0.78 -0.89, these results were acceptable. Regarding test-retest reliability, we found that the T-PSS-10 still demonstrated good reliability at a 4 week interval (0.83), which compares with the original findings that found test-retest reliability to be 0.85 in the college sample after 2 days and 0.55 in the community sample after 6 weeks[3].

### Additional limitations

Unlike the result reported by Cohen[3], the current study found that the relationships between T-PSS-10 and the validity criteria were unaffected by sex. A large-scale national probability study of the Thai population should be performed in order to confirm this result. Moreover, although the T-PSS-10 showed stability in the student sample over a 4-week period, limited re-test responses prevented such conclusions from being made in the clinical sample. Apart from convergent validity, discriminant validity requires further investigation in future studies.

## Conclusions

In summary, the T-PSS-10 is a reliable and valid instrument for estimating the level of stress associated with psychological or mental health problems within a Thai cultural context.

## Competing interests

The authors declare that they have no competing interests.

## Authors' contributions

Both authors conceived the study, developed the proposal, translated the PSS into Thai language, and wrote the manuscript. TW performed the statistical analysis. Both authors read and approved the final manuscript.
